# Modeling Early Stages of Trophectoderm–Endometrium Interactions Using Trophoblastic and Endometrial Organoids and the Generation of Lacunoids/Cystoids

**DOI:** 10.3390/cells14141051

**Published:** 2025-07-09

**Authors:** Islam M. Saadeldin, Budur Alshehri, Maha AlThubyani, Falah H. Almohanna, Goran Matic, Ayman A. Swelum, Serdar Coskun, Khalid A. Awartani, Abdullah M. Assiri

**Affiliations:** 1 Comparative Medicine Department, King Faisal Specialist Hospital and Research Centre, Riyadh 11211, Saudi Arabia; baalshehri@alfaisal.edu (B.A.); maha-althubyani@hotmail.com (M.A.); fmohanna@kfshrc.edu.sa (F.H.A.); gmatic@kfshrc.edu.sa (G.M.); assiri@kfshrc.edu.sa (A.M.A.); 2 College of Medicine, Alfaisal University, Riyadh 11533, Saudi Arabia; 3 King Fahd Medical City, Riyadh 11525, Saudi Arabia; serdar@kfshrc.edu.sa; 4 Department of Theriogenology, Faculty of Veterinary Medicine, Zagazig University, Zagazig 44519, Egypt; aymanswelum@zu.edu.eg; 5 Department of Pathology and Laboratory Medicine, King Faisal Specialist Hospital and Research Centre, Riyadh 11211, Saudi Arabia; 6 Department of Obstetrics and Gynaecology, King Faisal Specialist Hospital and Research Centre, Riyadh 11211, Saudi Arabia; kawartani@kfshrc.edu.sa

**Keywords:** trophoblast, organoids, lacunoids, cystoids, immunofluorescence, sheep

## Abstract

This study presents the first successful generation and comprehensive characterization of trophoblastic organoids (TOs) and the derivation of three-dimensional cavity- or sac-like structures—termed lacunoids/cystoids—from sheep intracytoplasmic sperm injection (ICSI) embryos. TOs were generated from sheep ICSI embryos for the first time and were shown to express trophoblastic markers at levels comparable to those in embryonic tissue. Detailed morphological characterization was conducted for both the TOs and the derived lacunoids/cystoids. Additionally, the TOs’ interactions with endometrial organoids (EOs), as well as those with preimplantation embryos, were investigated through co-culture experiments. The TOs expressed key trophoblastic markers, including CDX2, GATA3, syncytin-1, KRT18, KRT7, and Sox2, confirming their validity as a model for studying sheep trophoblast biology. The generation of lacunoids/cystoids from the TOs further revealed their structural and developmental characteristics, contributing valuable insights into early placental development and trophoblast-related pathologies. The TOs also supported extended embryonic development, and their co-culture with EOs induced dynamic changes in gene expression, particularly in angiogenesis-related genes, in both organoid types. This novel and reproducible in vitro model offers a reliable platform to study early placental development, effectively recapitulating the biological crosstalk between the trophectoderm and endometrium. The in-depth characterization of TOs and lacunoids/cystoids highlights their potential to advance our understanding of trophoblast differentiation and related developmental disorders.

## 1. Introduction

The trophoblast is a critical cell lineage in early mammalian development, playing a central role in implantation and placental formation. While trophoblastic organoids have been successfully derived from the placenta in several species, such as humans and mice, the generation of organoids from in vitro-produced embryos has not previously been explored. In vitro-produced embryo-derived trophectoderm provides a naïve form of trophoblasts that differ from placental trophoblasts in terms of their age and differentiation factors [1,2]. Saadeldin et al. [3] have reviewed the existing models for studying embryonic–maternal interactions using endometrial and trophoblast organoids in different species and showed that placenta, first-trimester chorionic villi, and trophoblastic stem cell-derived cells were the most common cells used for generating 3D trophoblastic organoids [2,4,5,6,7,8,9,10,11,12,13,14]. Furthermore, in vitro-produced embryo-derived trophoblasts provide an ethically accessible, scalable, and physiologically relevant platform that closely mimics epitheliochorial placentation, unlike human trophoblast organoids—which are constrained by ethical and tissue availability issues—and mouse models that differ significantly in their implantation timing and placental structure [15,16].

During the early stages of implantation, the embryo undergoes critical interactions with the maternal endometrium. A key structure that forms during this process is the lacunae system, which refers to fluid-filled small cavities that develop in the syncytiotrophoblast as the embryo invades the uterine lining. The lacunae play a significant role in establishing the foundations for the circulatory interface for maternal–fetal nutrient exchange and the development of the placenta [17,18,19]. While the structural development of lacunae is well-documented, the molecular pathways and signaling mechanisms guiding their formation remain incompletely understood.

The establishment of sheep trophoblastic organoids and lacunoids/cystoids from intracytoplasmic sperm injection (ICSI) embryos addresses a significant gap, paving the way for translational studies in reproductive biology and biotechnology. This work details the isolation, culture, and characterization of sheep trophoblastic organoids and lacunoids/cystoids, emphasizing their morphological and potential functional attributes.

## 2. Materials and Methods

### 2.1. Chemicals and Reagents

All chemicals used in this work were purchased from Sigma-Aldrich (St. Louis, MO, USA), unless otherwise specified.

### 2.2. Embryo Production via Intracytoplasmic Sperm Injection (ICSI)

Sheep ovaries were sourced from a local slaughterhouse in Riyadh and transported to the laboratory in a physiological saline solution (0.9% NaCl) supplemented with 1× antibiotic/antimycotic solution within four hours at 30–33 °C [20]. Cumulus–oocyte complexes (COCs) were aspirated with the aid of 18-gauge needles attached to 10 mL syringes, and oocytes with compact cumulus cell layers were selected and washed three times with a washing flushing medium (Ref# 10845060A, Origio, Måløv, Denmark). The COCs were matured in four-well dishes (Nunc™; Thermo Fisher Scientific, Waltham, MA, USA) using a commercial maturation medium (Ref# 82214010A, Medicult IVM^®^, Origio, Måløv, Denmark), 10% fetal bovine serum (Gibco, Thermo Fisher Scientific, Waltham, MA, USA), and 10 IU/mL each of human chorionic gonadotropin and pregnant mare serum gonadotropin for 24 h [21]. Mature COCs were harvested, and cumulus cells were removed using hyaluronidase. The oocytes were washed twice in the flushing medium.

Frozen semen straws were prepared according to Swelum et al. [22] and thawed at 37 °C. Spermatozoa were washed with PureSperm^®^ Wash medium (Ref# PSW-100, Nidacon, Mölndal, Sweden), and motile spermatozoa were purified through centrifugation for 15 min at 1500 rpm using discontinuous Origio^®^ gradients (40–80%, Ref# 84030060A, Origio). The spermatozoa were immobilized with polyvinylpyrrolidone 7% (*w*/*v* in HEPEs-HTF) (Ref# ART-4005, CooperSurgical^®^, Ballerup, Denmark). For the intracytoplasmic sperm injection (ICSI), using a micromanipulator (Eppendorf Piezoxpert^®^ micromanipulator, Eppendorf, Hamburg, Germany), a single immobilized sperm was aspirated into a glass injection pipette (5 µm) and directly injected into the cytoplasm of an in vitro-matured oocyte [23]. Injected oocytes were cultured in 25 μL of EmbryoMax Advanced KSOM Medium (Merck, MilliporeSigma, MA, USA) under mineral oil in a humidified atmosphere at 38.5 °C with 5% O_2_, 5% CO_2_, and 90% N_2_ for 7 days [24]. Blastocysts (day 8) were collected, and their zona pellucida was removed by means of incubation in 0.1% Pronase (*w*/*v* in PBS) for 30 s. Zona-free blastocysts were subsequently used for further experiments.

### 2.3. Isolation of Trophectoderm Cells

Trophectoderm cells were selectively isolated from day 8 sheep blastocysts (n = 10) through mechanical dissection and cultured in feeder-free culture conditions, as described in previous studies [25,26,27]. Four-well dishes were prepared by cooling them and adding 200 μL of diluted (0.1×) reduced growth factors and phenol-red-free Matrigel^®^ Matrix (Ref# 356231, Corning, Discovery Labware Inc., Bedford, MA, USA) to each well, followed by incubation at 37 °C for 30 min. After the excess Matrigel^®^ was removed, 500 μL of culture medium was added. The medium consisted of Dulbecco’s Modified Eagle Medium/Nutrient Mixture F-12, 10% fetal bovine serum (*v*/*v*), 0.1 mM β-mercaptoethanol, 1% nonessential amino acids (Invitrogen), and 1× Primocin. The trophectoderm cells were cultured in a humidified atmosphere with 5% CO_2_ at 38.5 °C. Between days 2 and 5 of culture, the embryos were monitored for attachment and outgrowth.

### 2.4. Derivation of Trophoblastic Organoids and Lacunoids/Cystoids from Trophectoderm Cells

After 8–10 days of trophectoderm outgrowths, the culture medium was aspirated and washed with PBS, trypsinized with 0.25 trypsin–EDTA, and harvested through centrifugation at 300× *g* for 2 min. Trophectoderm cell pellets were resuspended in cooled Matrigel^®^ and dispensed as 25 μL microdrops onto 4-well non-treated dishes (Nunc™; Thermo Fisher Scientific, Waltham, MA, USA). These Matrigel^®^ domes were incubated at 38.5 °C for 20 min to polymerize. The domes were then overlaid with trophoblast organoid culture medium (Table 1) and placed in a humidified incubator at 38.5 °C with 5% CO_2_. The domes were monitored daily, and the medium was replaced with a fresh organoid culture medium every three days. Bright-field microscopy was used to analyze the 3D structure of the resultant organoids and derivatives.

### 2.5. Derivation of Trophoblastic Organoids and Lacunoids/Cystoids from ICSI Blastocysts

Individual zona-free day 8 blastocysts (n = 14) were washed in PBS, mechanically cut to remove the inner cell mass, and placed in 50 μL of 0.25 trypsin–EDTA for 1 min at 37 °C with gentle pipetting. The reaction was stopped by adding 100 μL of trophoblast medium, and the cells were then harvested by means of centrifugation. Embryonic cells were resuspended in 30 μL cooled Matrigel^®^, dispensed as 15 μL microdrops onto 4-well non-treated dishes, and handled the same as previously described. Lacunoid/cystoid formation was observed daily.

### 2.6. Generation of Endometrial Organoids (EOs)

Female sheep uteri (n = 8) with no apparent pathological lesions were sourced from a local slaughterhouse in Riyadh and transferred to the laboratory within 2 h in an icebox. Sheep endometrial organoids were generated using a procedure adapted from Saadeldin et al. [14] with modifications. The uterine horns were dissected and rinsed with phosphate-buffered saline (PBS) containing 75 μg/mL kanamycin. The endometrial layers were excised, placed in Petri dishes, and cut into 1 mm fragments. These pieces were washed three times with PBS using 100 μm strainers and digested with 0.01% collagenase type I at 38 °C for 2 h with intermittent shaking. The resulting cell suspensions were filtered through 70 μm strainers and centrifuged at 450× *g* for 3 min at room temperature. After three PBS washes, the purified cell pellets were resuspended in cooled Matrigel^®^ and dispensed as 30 μL droplets onto Petri dishes. The droplets were incubated at 38 °C for 20 min to solidify, then overlaid with organoid culture medium and incubated at 38.5 °C in a 5% CO_2_ atmosphere.

The organoid culture medium was replenished every three days and consisted of advanced DMEM/F12, N2 supplement, and B27 supplement (without vitamin A), each at 1× concentration. Additional ingredients included Primocin (100 μg/mL), N-acetyl-L-cysteine (1.25 mM), and L-glutamine (2 mM). Growth factors like recombinant human EGF (50 ng/mL), Noggin (100 ng/mL), R-spondin-1 (500 ng/mL), FGF-10 (100 ng/mL), and HGF (50 ng/mL) were incorporated. The medium also contained ALK-4, -5, -7 inhibitor (500 nM), nicotinamide (10 mM), β-estradiol (10 nM), progesterone (1 μM), and 8-bromoadenosine 3′,5′-cyclic monophosphate (1 μM). The hormonal additives included prolactin (20 ng/mL), human placental lactogen (1 μg/mL), and human chorionic gonadotropin (1 μg/mL), as described previously [14]. For passaging, the culture medium was removed, and the Matrigel^®^ domes were dissociated by means of pipetting and centrifugation at 450× *g* for 3 min at 4 °C. The organoids were treated with 0.05% trypsin–EDTA solution, gently pipetted, and incubated at 38 °C for 4 min. Enzymatic activity was stopped by adding PBS with 0.1% bovine serum albumin (BSA), and the cells were collected through centrifugation at 450× *g* for 3 min. The cell pellets were then subcultured at a 1:2 ratio, following the established protocol.

### 2.7. Immunofluorescence Characterization of Trophectoderm, Trophoblastic Organoids, Endometrial Organoids, and Lacunoids/Cystoids

Immunofluorescence staining was carried out for CDX2, GATA3, syncytin, β-catenin, Sox2, KRT7, KRT18, lamin, vimentin, VEGFA, VEGFR1, and Ki-67 following a modified version of a previously described protocol [14]. Trophectoderm monolayers, trophoblastic organoids (TOs), lacunoids/cystoids, or endometrial organoids (EOs) (n = 20–25) were fixed with 4% paraformaldehyde, rinsed with PBS, and permeabilized using 0.1% Triton-X100. Blocking was achieved with 1% goat serum for 30 min at room temperature. The primary antibodies included CDX2 (mouse IgG, AM392-5M, Biogenex, Fremont, CA, USA), GATA3 (mouse IgG, E2N1Y, 96098, Cell Signaling, Danvers, MA, USA), Syncytin-1 (ERVW-1) (rabbit IgG, BS-2962R, ThermoFisher, Waltham, MA, USA), beta-catenin (rabbit IgG, [E247] (ab32572, Abcam, Cambridge, UK)), Ki67 (SolA15) (rat IgG, 14-5698-82, Bioscience™, Invitrogen, Carlsbad, CA, USA), Sox2 (mouse IgG, MAB4343; EMD MilliporeSigma, Burlington, MA, USA), cytokeratin KRT7 (mouse IgG, RCK105, ab9021, Abcam), cytokeratin KRT18 (mouse IgG, MA1-06326, ThermoFisher), lamin (636) (mouse IgG, sc-7292, Santa Cruz Biotech., Dallas, TX, USA), VEGFA (rabbit IgG, ab46154, Abcam), and VEGFR1 [Y103] (rabbit IgG, ab32152, Abcam). The cells were incubated with these primary antibodies diluted in 1% goat serum (dilution 1:100 (*v*/*v*), according to the manufacturer’s instructions) for 2 h at 37 °C, followed by three PBS washes. Secondary antibodies (diluted 1:200 in PBS) were then applied, including goat anti-mouse Alexa Fluor^®^ 594 (ab150116, Abcam), goat anti-mouse Alexa Fluor^®^ 488 (ab150117, Abcam), goat anti-rabbit Alexa Fluor^®^ 488 (ab150077, Abcam), and goat anti-rat Alexa Fluor^®^ 488 (ab150165, Abcam), and incubated for 1 h at 37 °C. To visualize the cell membranes in lacunoids/cystoids, a deep red CellMask™ plasma membrane stain (ThermoFisher) was used. Afterward, the cells or organoids were washed three times with PBS, and nuclei were counterstained with VECTASHIELD^®^ antifade mounting medium containing 4′,6-diamidino-2-phenylindole (DAPI) for 5 min while mounted on glass microscopic slides. VEGFR2/KDR/Flk-1 was stained directly with fluorescein-conjugated antibodies (R&D Systems, Bio-Techne|FAB357F) for 2 h and counterstained with DAPI. Fluorescence imaging was conducted using an inverted microscope (Olympus IX71, Olympus, Tokyo, Japan) connected to an epifluorescence unit (Olympus U-RFL-T, TH4-200, Olympus) and a digital camera (C13440, Hamamatsu, Saitama, Japan). Confocal microscopy was performed through an Evident FLUOVIEW™ FV4000 confocal laser scanning microscope (Evident Scientific, Olympus). UV excitation was applied at 488 nm for green fluorescence, at 568 nm for red fluorescence, and at 649 nm for magenta fluorescence. To rule out autofluorescence and nonspecific secondary antibody binding, control experiments omitting the primary antibodies (no primary control) were performed with identical incubation times.

### 2.8. Effects of Co-Culturing Trophoblastic Organoids with Endometrial Organoids

Passages No. 2 of both TOs and EOs were allocated into 4-well dishes in separate 30 μL Matrigel domes with an average distance of 1–2 mm between the domes. Each dome contained an average of 12.3 ± 2.5 organoids and was covered with the same culture medium used for TO culture. Morphological features were recorded daily, and the immunofluorescence of both the TOs and EOs (n = 25 each) was examined on day 6 of the co-culture. Individual TOs and EOs were sampled for total RNA extraction.

### 2.9. Effects of Co-Culturing Trophoblastic Organoids with Day 8 Blastocysts with or Without Attachment to Matrigel Domes

To explore the effects of TOs on embryo development, day 8 blastocysts (n = 10) were co-cultured with TO domes, aligned to either attach or not attach to the Matrigel domes. Control embryos (n = 10) were cultured in the same medium without the presence of organoids. Embryo development was monitored for 12 days, and morphological changes were recorded daily. Each embryo’s diameter was measured at least four times, and all measurements were documented. The morphometric parameters of these images were subsequently analyzed using ImageJ 1.54p software (NIH, Bethesda, MA, USA).

### 2.10. Real-Time Polymerase Chain Reaction (RT-qPCR)

Total RNA was isolated from embryos (n = 5), TOs (n = 25, cultured with or without EOs), EOs (n = 25, cultured with or without TOs), and endometrium 2D cultures (3 replicates). The RNeasy Mini Kit (Qiagen, Hilden, Germany) was used for RNA extraction. The RNA concentration and purity were assessed using a NanoDrop™ 2000/2000c spectrophotometer. Complementary DNA (cDNA) was synthesized using the SuperScript III First-Strand Synthesis System following the manufacturer’s protocol. Briefly, the reaction mixture included RNA (10–20 ng per reaction), random hexamers (50 ng/µL), 10X RT buffer (2 µL per reaction), 25 mM MgCl2 (4 µL per reaction), 0.1 M DTT (2 µL per reaction), 10 mM dNTP mix (1 µL per reaction), SuperScript III RT (200 U/µL, 1 µL per reaction), and RNaseOUT Recombinant RNase Inhibitor (1 µL per reaction). Relative quantitative PCR was performed using PowerUp SYBR Green (ThermoFisher) to amplify the target cDNA with specific primers (Table S1). The PCR cycling conditions consisted of an initial denaturation cycle at 95 °C for 2 min, followed by 40 cycles of denaturation at 95 °C for 15 s, annealing at 60 °C for 30 s, and extension at 72 °C for 1 min. A final extension step at 72 °C for 5 min was performed. A melt curve analysis was conducted post-amplification to verify the reaction specificity and check for primer dimers. All PCR reactions were performed in triplicate for technical consistency [24]. The relative quantification (RQ) of the target transcripts was determined by comparing them to the housekeeping genes GAPDH and ACTB in day 7 embryos, using the 2^−ΔΔCT^ method.

### 2.11. Statistical Analysis

To compare embryo growth, the data were analyzed with Student’s *t*-test, and *p* < 0.05 was considered to indicate a significant difference. For the RT-qPCR data, the retrieved values were used to calculate the mean and the standard error of the mean (SEM). To assess differences between groups, a one-way analysis of variance (ANOVA) was conducted. When a significant difference was detected (*p* < 0.05), Tukey’s Honest Significant Difference (HSD) test was employed as a post hoc analysis to verify specific group comparisons.

## 3. Results

### 3.1. Successful Derivation of Trophoblastic Organoids

**Figure 1:**
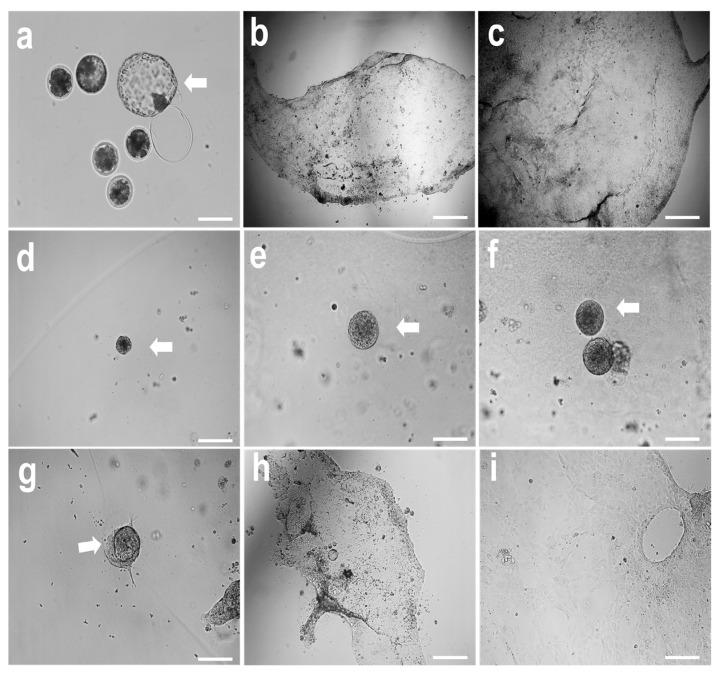
Establishment and plasticity of trophoblast organoids (TOs) from trophectoderm outgrowths. (**a**–**c**) Day 8 hatched blastocysts ((**a**), white arrow) were cultured under feeder-free culture conditions, and trophectoderm outgrowths were observed ((**b**,**c**)). Trophectoderm cells were trypsinized and cultured in Matrigel domes. Solid organoids were observed with a progressive increase in size ((**d**–**f**), white arrows). (**g**–**i**) Images show the plasticity of the TOs and reformation of the trophoblast monolayer after seeding on tissue culture dishes ((**g**), white arrow). Scale bar = 200 µm. (**a**,**d**,**g**) are pictures on day 1 of culture; (**b**,**e**,**h**) are pictures on day 6 of culture; (**c**,**f**,**i**) are pictures on day 12 of culture.

Trophoblast cells derived from ICSI embryos proliferated in a monolayer (Figure 1a–c). After their trypsinization and culture in Matrigel, they grew and developed efficiently in 3D culture systems and displayed compact, spheroid structures named trophoblastic organoids (TOs) (Figure 1e,f). These organoids showed plasticity and were capable of reforming trophoblast monolayer outgrowths after being transferred from the Matrigel and culture onto tissue culture dishes (Figure 1g–i).

### 3.2. Morphological and Structural Features of Trophoblastic Organoids and Lacunoids/Cystoids

**Figure 2:**
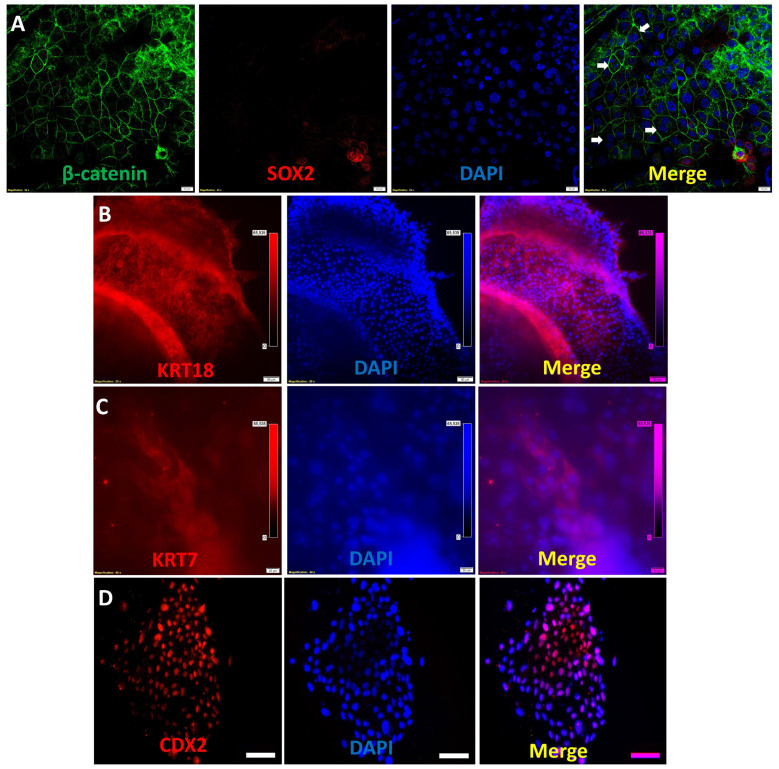
Immunofluorescence of sheep trophectoderm outgrowths. Trophectoderm outgrowths were double-immunostained against β-catenin and SOX2 (**A**) (scale bar = 20 µm) and single-immunostained against KRT18 (**B**) (scale bar = 50 µm), KRT7 (**C**) (scale bar = 20 µm), and CDX2 (**D**) (scale bar = 50 µm). White arrows in Panel A indicate the presence of giant binucleated trophoblasts. Nuclei were counterstained with DAPI, and merged pictures were generated using ImageJ 1.54p software.

Confocal and immunofluorescence microscopy confirmed the presence of trophectoderm markers β-catenin, SOX2, cytokeratin-18 (KRT18), cytokeratin-7 (KRT7), and CDX2 in the monolayer culture (Figure 2, Video S1).

**Figure 3:**
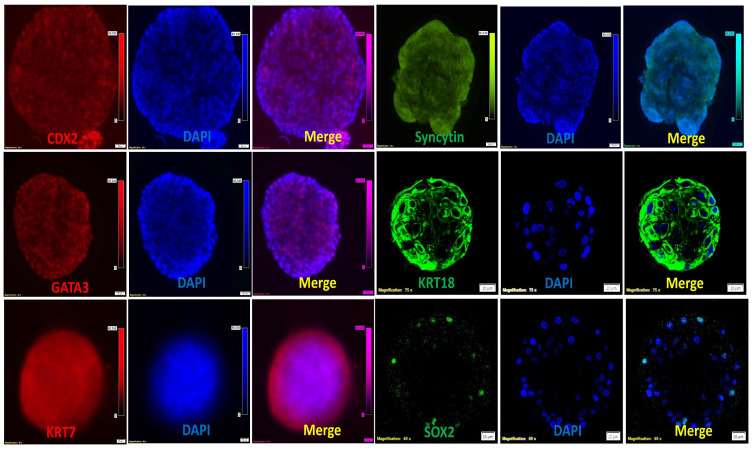
Immunofluorescence of single sheep trophoblastic organoids. Trophoblastic organoids (day 7) were immunostained against CDX2 (scale bar = 50 µm), syncytin (scale bar = 100 µm), GATA3 (scale bar = 50 µm), KRT18 (scale bar = 20 µm), KRT7 (scale bar = 20 µm), and SOX2 (scale bar = 20 µm). Nuclei were counterstained with DAPI, and merged pictures were generated using ImageJ 1.54p software.

Expression was conserved in the 3D structural organization of the trophoblastic organoids (TOs), besides the expression of the trophoblastic markers CDX2, GATA3, and syncytin-1 (Figure 3).

**Figure 4:**
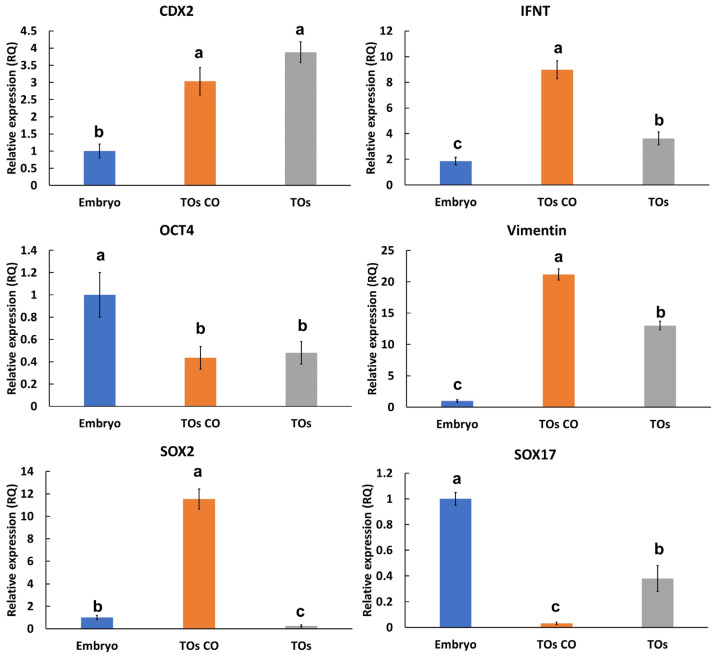
Relative quantification of transcripts in embryos and TOs by means of qPCR. Three replicates of day 7 blastocysts (n = 5) and TOs cultured with or without EOs (n = 25 each) were used to generate the data. Data are presented as means ± SEMs and were compared with a one-way ANOVA and Tukey’s HSD test. Bars carrying different letters (a, b, and c) are statistically different at *p* < 0.05.

The qPCR results showed increased expression of CDX2, interferon tau (IFNT), and vimentin transcripts in the resulting TOs at levels of 3.9-, 2.2-, and 12.2-fold, respectively, when compared with their embryonic expression (Figure 4). Oct4, SOX2, and SOX17 were significantly reduced to 0.5-, 0.25-, and 0.35-fold, respectively, when compared with their embryonic expression (Figure 4).

**Figure 5:**
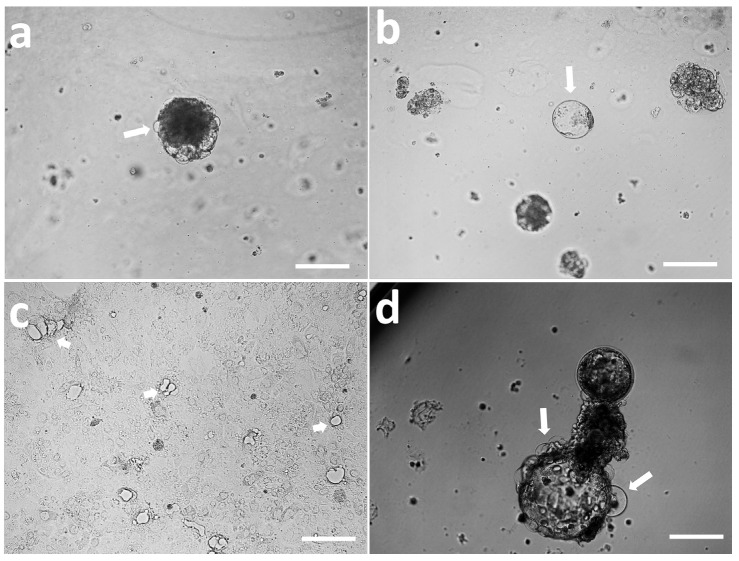
Trophoblastic lacunoids/cystoids (white arrows) observed to protrude from trophoblastic organoid cultures (**a**) or float in Matrigel domes (**b**), within trophectoderm cultures (**c**) and extended embryo cultures (**d**). Scale bar = 100 µm.

In the late culture of TOs (day >12), some cystic structures protruded from the TOs (Figure 5a); these structures were individually observed in the Matrigel domes as well (Figure 5b). These three-dimensional cavity- or sac-like structures that form in TOs are named lacunoids/cystoids. This finding coincides with the observations from long-term cultures of trophoblasts in monolayers (Figure 5c) and embryos (Figure 5d). We named these 3D structures trophoblastic lacunoids/cystoids (TLCs).

**Figure 6:**
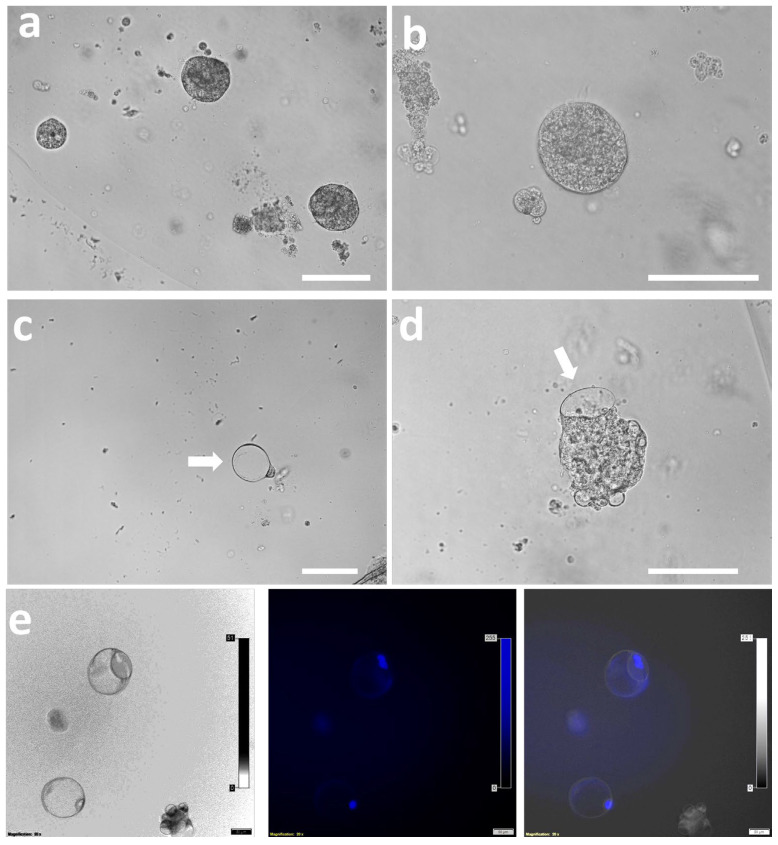
The persistence of both trophoblastic organoids and lacunoids/cystoids after passaging of the trophoblastic organoids. TOs maintained their compact 3D morphology in passages 1 and 2 ((**a**) and (**b**), respectively). On days 6–10 of culture, lacunoids/cystoids appeared, floating in the Matrigel ((**c**), white arrow) dome or protruding from the TOs ((**d**), white arrow). Scale bar = 100 µm. (**e**) Lacunoids/cystoids can be observed in mono- or binucleated cells after live staining with Hoechst stain (scale bar = 50 µm) and either monopolar or bipolar (more images are presented in Figure S1).

Similar patterns were observed after organoid trypsinization and passaging (Figure 6a–d); TOs and TLCs maintained their morphology. Lacunoids/cystoids exhibited a hollow, lumen-containing morphology. Live-cell imaging after nuclear staining with bisbenzimide (Hoechst 33342, 1 mg/mL) confirmed polar or apically localized cells (Figure 6e).

**Figure 7:**
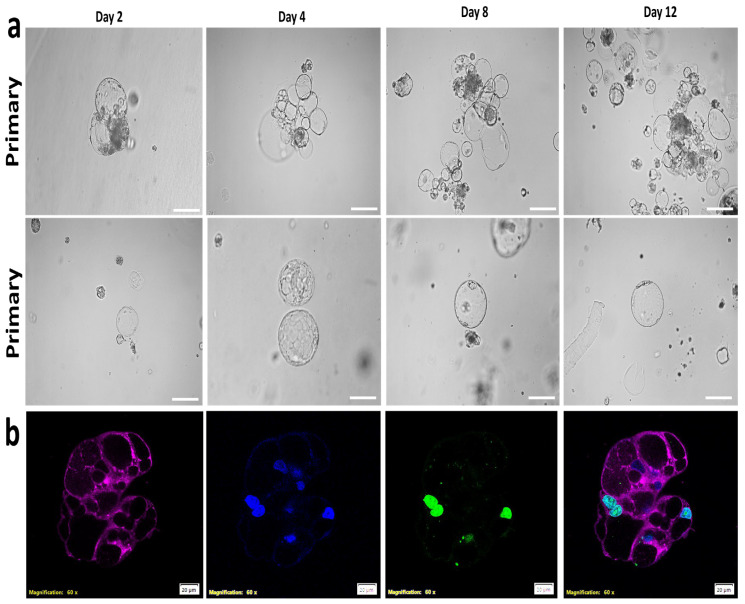
Generating trophoblastic lacunoids/cystoids through embryo trypsinization, passaging, and development for 12 days (**a**). Scale bar = 100 µm. (**b**) Immunofluorescence of trophoblastic lacunoids/cystoids for proliferation marker Ki-67 and cytoplasmic membrane staining with deep-red stain. Scale bar = 20 µm.

These TLCs can be passaged and maintain their morphology (Figure 7a), with proliferating nuclei expressing Ki-67 (Figure 7b).

### 3.3. Effects of Trophoblastic Organoids on Extended Embryo Culture

**Figure 8:**
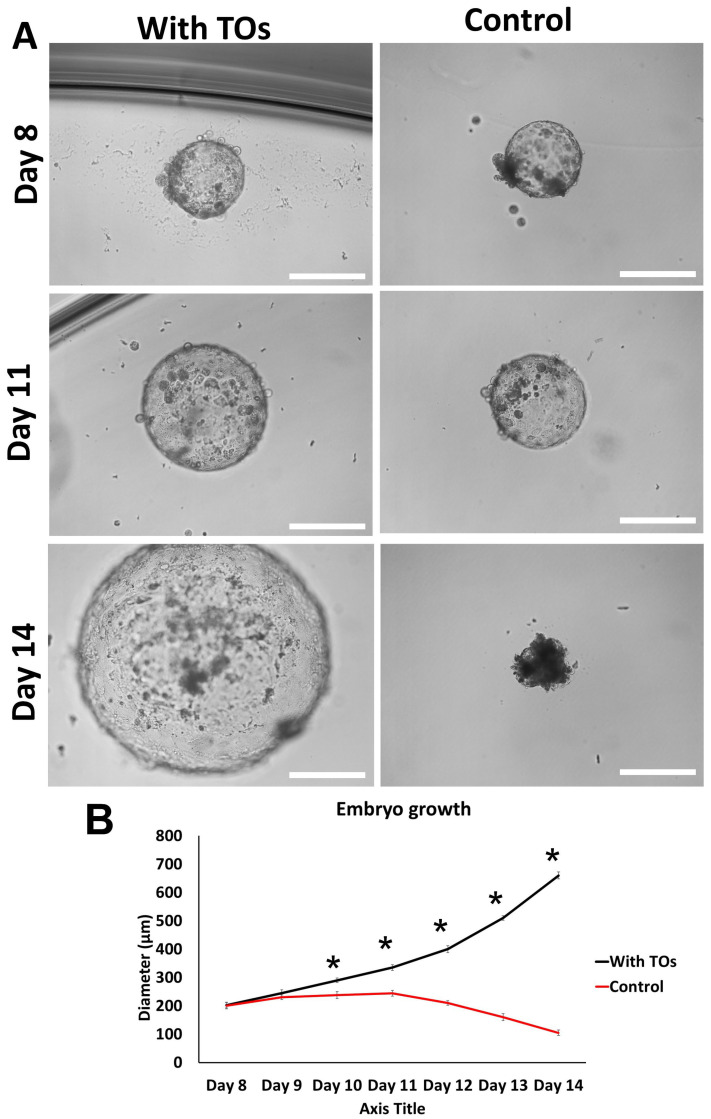
Effects of day 7 blastocyst co-culture with or without trophoblastic organoids. (**A**) The images show blastocyst development throughout 7 days of culture with or without TOs. Scale bar = 200 µm. (**B**) The graph analysis shows the means (±SEMs) of daily embryo diameter as an indicator of growth from day 8 until day 14. The data (n = 10 per group) were analyzed using Student’s *t*-test, and asterisks (*) indicate significant differences between the two groups on a daily basis.

Embryos (n = 8) co-cultured in medium together with TO domes were capable of a progressive increase in diameter and extended culture till day 14, while the control embryos that were cultured in the same conditions without the presence of TCs showed signs of degeneration on day 10 of culture (Figure 8A,B). The culture was terminated intentionally because of the complete degeneration of the control group on day 14.

### 3.4. Characterization of Sheep Endometrial Organoids

**Figure 9:**
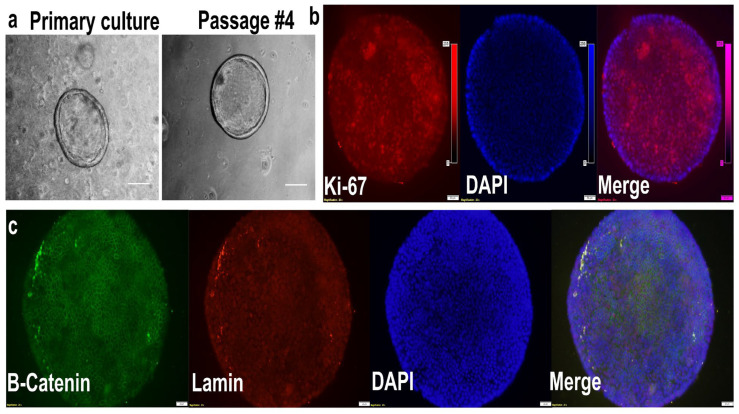
Characterization of endometrial organoids through bright-field microscopy and double immunostaining. Bright-field images (**a**) show the generation and maintenance of the culture of endometrial organoids from sheep (*Ovis aries*) in primary culture and after trypsinization and subculture (passage 4). Scale bar = 100 µm. For immunofluorescence, endometrial organoids were targeted with primary antibodies against Ki-67 (nuclear) ((**b**), scale bar = 50 µm), β-catenin (cytoplasmic), and lamin (nuclear) ((**c**), scale bar = 20 µm). Green fluorescence was visualized at 488 nm and red fluorescence was visualized at 594 nm, while DAPI was visualized at a 358 nm ultraviolet wavelength.

By day 4, the endometrial cells had reorganized and formed small, 3D, spherical, multicellular organoids, which continued to grow until day 7 (Video S2). The organoids expanded in size over this period, measuring 40–60 µm in diameter on day 4 and increasing to 200–250 µm by day 7 (Figure 9a). Their morphology remained consistent through four passages, even after being transferred into fresh Matrigel^®^ domes following trypsinization (Figure 9a). An immunofluorescence analysis showed that Ki-67, a proliferation marker (Figure 9b), and lamin (Figure 9c), a nuclear envelope structural protein, were localized in the nucleus; beta-catenin, a cell adhesion protein, was localized in the cytoplasm (Figure 9c).

### 3.5. Interaction of Embryos with Trophoblastic Organoids and Endometrial Organoids

**Figure 10:**
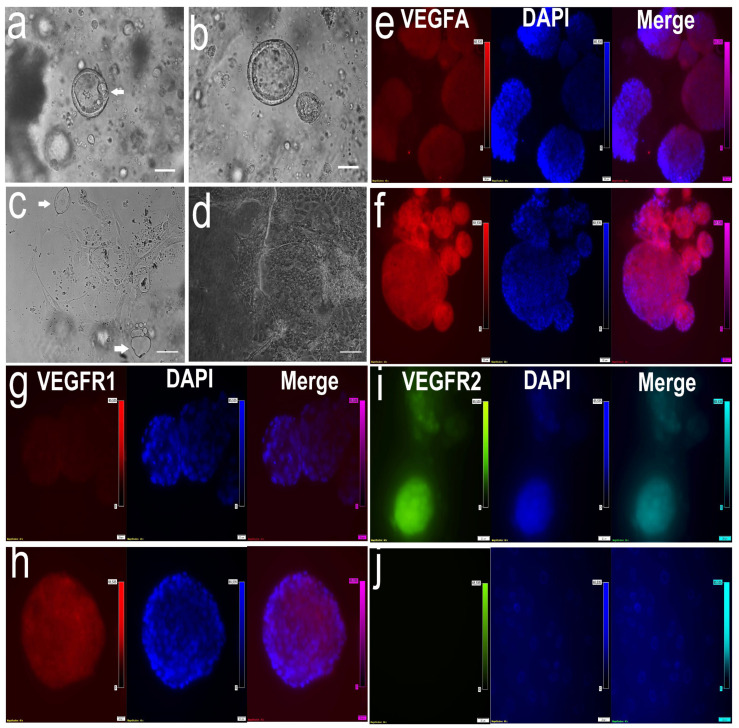
Coculture of trophoblastic organoids (TOs) with endometrial organoids (EOs) in separated domes overlaid with the same culture medium. (**a**,**b**) Cystic structures (white arrows) appeared in the wall of the day 5 EOs (**a**) but were not observed in the control group (**b**) (more images are shown in Supplementary Figure S2). (**c**,**d**) When EOs were cultured in a TO-conditioned medium, attached to the tissue culture dish, and grew into a 2D monolayer, the cystic structures were also observed (**c**, white arrows) when compared to the control (**d**). Scale bar = 100 µm. Pictures e-j are the immunofluorescence of angiogenesis-related factors in the TOs and EOs: ((**e**) and (**f**)) VEGFA expression in TOs and EOs, respectively; ((**g**) and (**h**)) VEGFR1 absence in TOs and expression in EOs, respectively; ((**i**) and (**j**)) VEGFR2 expression in TOs and absence in EOs, respectively. Scale bar in (**e**–**j**) = 20 µm.

When TOs were co-cultured with endometrial organoids (EOs) in separate domes within the same culture medium, lacunoids/cystoids appeared on both TO and EO domes after 36 h of culture (Figure 10a,b). These structures were not observed during the entire culturing and passaging of the EOs alone. Endometrial outgrowths resulting from culturing EOs on culture dishes showed the appearance of lacunoids/cystoids as well (Figure 10c,d). Immunofluorescence showed the expression of VEGFA in both the TOs and EOs (Figure 10e,f), VEGFR1 in the EOs (Figure 10g,h), and VEGFR2 in the TOs (Figure 10i,j).

**Figure 11:**
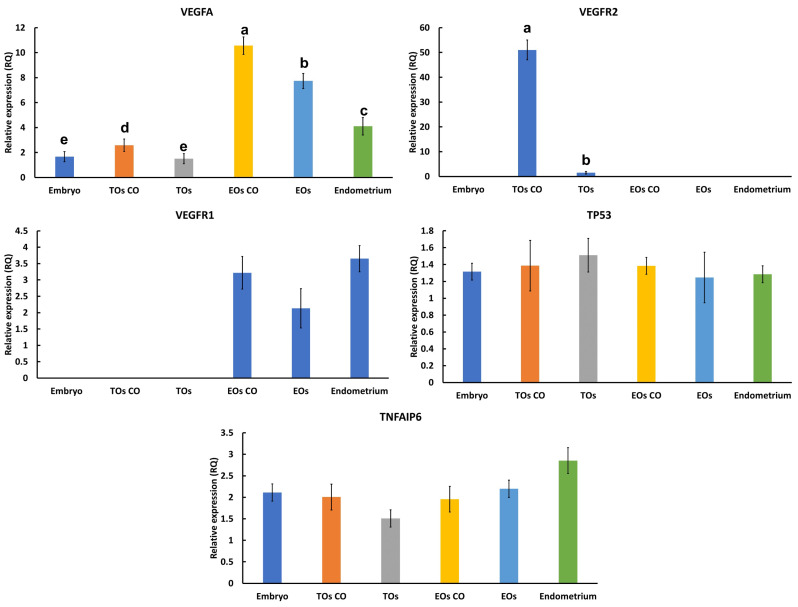
Relative quantification of transcripts in TOs, EOs, embryos, and endometrium by means of qPCR. Three replicates of endometrium monolayers, day 7 blastocysts (n = 5), and TOs cultured with or without EOs (n = 25 each) were used to generate the data. Data are presented as means ± SEMs and were compared via a one-way ANOVA and Tukey’s HSD test. Bars carrying different letters (a, b, c, d, and e) are statistically different at *p* < 0.05.

The qPCR data confirmed the expression of VEGFA in both TOs and EOs, VEGFR1 in EOs, and VEGFR2 in TOs (Figure 11), with no effects on apoptosis-related signals (TP53 and TNFAIP6). VEGFA showed an increase in expression in TOs co-cultured with EOs and a similar increase in EOs co-cultured with TOs. Additionally, VEGFR2 showed increased expression in TOs co-cultured with EOs, while VEGFR1 expression in EOs showed no difference when they were co-cultured with TOs.

**Figure 12:**
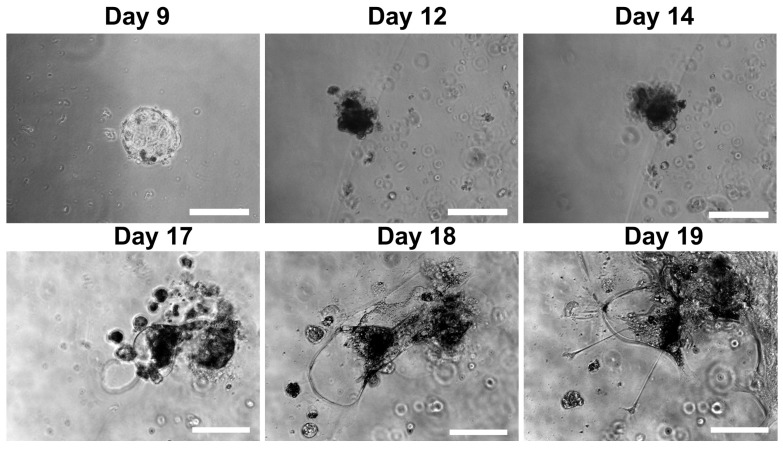
Trophoblastic lacuna formation when embryos were attached to Matrigel and co-cultured with endometrial organoids. Scale bar = 200 µm for days 9–14, 100 µm for days 17–19.

Interestingly, IFNT and vimentin showed 2.6- and 1.6-fold increases in expression in TOs co-cultured with EOs, while CDX2 and OCT4 expression in TOs showed no difference when TOs were co-cultured with EOs (Figure 4). Paradoxically, SOX2 showed a 40-fold increase in its expression in TOs co-cultured with EOs, while SOX17 showed a 10-fold decrease in its expression in TOs co-cultured with EOs (Figure 4). Furthermore, embryos (day 8) co-cultured with EOs aligned to attach to Matrigel showed lateral attachment of the embryos and the formation of cysts or lacunae on the 4th day; these lacunae grew and became clearer on the 10th day. After that, the embryonic parts attached to the Matrigel propagated and formed roots of trophoblastic cells with differentiated elongated cytoplasmic protrusions (Figure 12).

## 4. Discussion

This study highlights the first trial resulting in the successful derivation and characterization of sheep trophoblastic organoids and lacunoids/cystoids. Trophoblastic lacunoids/cystoids (TLCs) refer to cavity- or sac-like three-dimensional structures generated in trophoblastic organoid models that resemble early developmental spaces (like lacunae). These structures offer a paradigm to simulate aspects of early embryo implantation, including fluid-filled cavities that are important for nutrient exchange, tissue invasion, and vascularization between the trophectoderm and the endometrium during the establishment of pregnancy.

The use of ICSI embryos offers a controlled system for generating trophoblastic structures, ensuring genetic uniformity and reproducibility. The findings also underscore the role of angiogenesis signaling pathways in trophoblast differentiation and morphogenesis, with implications for enhancing reproductive efficiency in livestock and understanding placental disorders.

TLCs express trophoblast-specific proteins, supporting the successful derivation of these cells from the trophectoderm. For instance, CDX2, a member of the caudal-related homeobox transcription factor family, plays a vital role in defining and preserving trophoblast identity. Prior research underscored its importance in controlling the destiny and development of trophoblast stem cells [28]. GATA3, a zinc finger transcription factor, is also critical for trophoblast differentiation and function. It governs the expression of key genes involved in trophoblast lineage commitment and helps establish trophoblast-specific gene expression profiles [29]. Syncytin proteins, originating from retroviral envelope genes incorporated into the mammalian genome, serve a distinctive function in trophoblast physiology. They promote cell–cell fusion events essential for the formation of the syncytiotrophoblast, a multinucleated layer vital for nutrient exchange and hormone production in the placenta [30]. Besides these, cytokeratins 7 and 18 [31,32] and β-catenin [33] are essential for trophoblast functions. In particular, KRT7 participates in forming the cytotrophoblasts, which are required for implantation [32]. SOX2 was found in TOs with a few stained nuclei and low expression through qPCR compared to its embryonic expression, which reflects its initial participation in trophectoderm formation during the preimplantation embryonic stages [34]. Interestingly, SOX2 expression showed an increase in TOs co-cultured with EOs, suggesting a role in the interaction between trophoblasts and endometrium. Conversely, SOX17 promotes differentiation toward the definitive endoderm and contributes to extraembryonic development, signaling the transition from pluripotency to lineage commitment, and is expected to be reduced during trophoblast establishment [35]. Moreover, TOs highly express interferon tau (IFNT), which is specific to ruminants’ trophoblasts during the preimplantation stage (days 12–13). Its expression was multiplied when TOs were co-cultured with EOs because of its involvement in the maternal recognition of pregnancy and supporting uterine receptivity [24,36,37,38,39]. On the other hand, TOs express a lesser amount of OCT4 (POU5F1), which is a master regulator of pluripotency, maintaining the undifferentiated state of the inner cell mass; its expression is known to be temporally reduced with trophoblast derivation [24].

It has been revealed that human first-trimester trophoblast cell lines and trophoblast stem cells express and release VEGF, VEGFR1, and VEGFR2 in spent culture medium [40,41], leading to speculation on their roles in increasing angiogenesis in the early stages of implantation [42,43]. Moreover, trophoblasts secrete VEGFA in an autocrine manner to maintain their survival in hypoxic conditions [44]. VEGF is essential in modulating trophoblast giant cell development and differentiation to maintain the homeostasis of the maternal vascular spaces in mouse placenta [45]. Similarly, VEGF expression was observed in both cytotrophoblasts and endometrium at the lacunar stage of implantation in rhesus monkeys [46]. Our results showed that both endometrium and TOs express VEGFA, and this expression significantly increased when both organoids were co-cultured together. While VEGFR1 was expressed in TOs and VEGFR2 was expressed in EOs, their expression was significantly multiplied under co-culture conditions. This differential expression suggests specific roles for each VEGF receptor and VEGFA in stimulating complementary angiogenesis through autocrine and paracrine crosstalk between the maternal and embryonic sides. However, further research is required to elucidate these mechanisms, as well as their interplay with other angiogenesis-related signals such as Wnt/β-catenin [47,48] and HIF-1α [49,50].

Cystic protrusions, known as vacuolated cells, were noted in several previous reports and claimed to be a sign of apoptosis in embryos [51,52,53,54]; however, there was no evidence for or against this, until now. Apoptosis typically takes place in isolated single cells. While the entire process is estimated to last between 12 and 24 h, noticeable morphological changes in cell cultures are completed in under two hours [55]. Our results showed no difference in the expression of the apoptosis- and necrosis-related genes TP53 and TNFAIP6 [56,57,58,59]. Our results contradict a long-lived hypothesis regarding signs of apoptosis in embryos and support the presence of cyst-forming cells (lacunoids/cystoids) in both embryos and embryo-derived cells. These lacunoids/cystoids replicate and increase the size of the cysts over time. Our results align with the findings of recent reports [32,60,61,62] that noted lacuna formation on the peripheral parts of trophoblasts in different models (Table 2).

Interestingly, the vacuoles were previously observed in trophoblasts at the time of implantation in rats [63,64], mice [65], humans [66], macaques [17,67], and marmosets [68]. Similar spaces or voids were found at the implantation sites on day 18 of pregnancy in sheep, with no further characterization or explanation of their origin [69]. Further research is required to elucidate the physiological involvement of these TLCs and to determine how normal lacunae appear in temporal and spatial terms, their correlation with angiogenesis, and how abnormal lacuna formation correlates with pregnancy disorders. Moreover, the role of environmental and maternal factors, such as stress, toxins, endocrine disruptors, or nutrition, on lacuna development is still an underexplored area.

Understanding the formation of trophoblast-regulated structures, including lacunae, shifts our perspective on early placental development. It suggests potential implications for placental pathologies, such as impaired lacuna formation potentially signaling deficient trophoblast function, contributing to early pregnancy loss or abnormal implantation (e.g., ectopic pregnancy, placenta previa). Additionally, this perspective opens avenues for exploring novel therapeutic targets and potential biomarkers. Modulating VEGFA or other signaling pathways involved in trophoblast regulation could offer strategies for correcting defective placentation or managing conditions like preeclampsia and placenta accreta spectrum disorders. The expression levels of VEGFA, VEGFR, and other factors in early pregnancy may also serve as early indicators of trophoblast behavior and implantation success, guiding clinical interventions and monitoring.

## 5. Conclusions

Trophoblastic organoids recapitulate key aspects of trophoblast morphology and function, providing a valuable platform to study placental development, maternal–fetal interactions, and reproductive pathologies. The formation of a lacunoid/cystoid model offers a paradigm for advancing research on the early stage of placental biology, trophoblast invasiveness, angiogenesis, and nutrient exchange with maternal tissues, as well as providing insights into species-specific developmental processes.

## Figures and Tables

**Table 1:** Composition of trophoblastic organoid culture medium.

Component	Source	Catalog Number	Working Conc.
Advanced DMEM/F12	Life Technologies, Carlsbad, CA, USA	12634010	1X
ALK-4, -5, -7 inhibitor (A83-01)	System Biosciences, Palo Alto, CA, USA	ZRD-A8-02	500 nM
B27 supplement minus vitamin A	Life Technologies	12587010	1X
L-glutamine	Life Technologies	25030-024	2 mM
N2 supplement	Life Technologies	17502048	1X
N-Acetyl-L-cysteine	Sigma-Aldrich	A9165-5G	1.25 mM
Nicotinamide	Sigma-Aldrich	N0636	10 nM
Primocin	Invivogen, San Diego, CA, USA	ant-pm-1	100 μg/mL
Recombinant human EGF	Peprotech, Cranbury, NJ, USA	AF-100-15	50 ng/mL
Recombinant human FGF-10	Peprotech	100-26	100 ng/mL
Recombinant human HGF	Peprotech	100-39	50 ng/mL
Recombinant human Noggin	Peprotech	120-10c	100 ng/mL
Recombinant human Rspondin-1	Peprotech	120-38	500 ng/mL
Rock inhibitor (Y-27632)	Sigma-Aldrich	Y0503	10 µM

**Table 2:** Evidence of lacuna observations using different in vitro models.

Species	Model	Major Finding	Ref.	Notes
Human	Synthetic embryo model (blastoids)	Multiple cavities between the outer sides of a syncytiotrophoblast-like layer	[60]	On day 6 of blastoid formation
Human	IVF-derived embryo	Cavities in the outer layer of attached trophoblasts	[61]	Day 11
Human	Human first-trimester trophoblast cell spheroid outgrowth	Cavities central to spheroid attachment and at the periphery	[62]	Cultured 3D spheroids forming 2D outgrowths
Human	Trophoblast-enriched cell (villi from first-trimester placental tissue)	Lacunae present within the syncytial areas	[2]	Similarly to Carnegie stage 5b embryos (approximately 9 days after fertilization)
Marmoset	Trophoblast stem cell differentiation in vitro	Peri-implantation trophoblast-like stem cells form floating vesicles at the periphery	[32]	Day 5 of floating after passaging

## Data Availability

All data are provided in the manuscript and Supplementary Files.

## Supplementary Materials

The following supporting information can be downloaded at https://www.mdpi.com/article/10.3390/cells14141051/s1, Table S1: Primers used for RT-qPCR analysis; Figure S1: Different forms of floating lacunoids/cystoids observed by live staining (scale bar = 20 µm) and brightfield microscopy (scale bar = 100 µm); Figure S2: Different forms of lacunoids/cystoids observed in endometrial organoids when co-cultured with trophoblastic organoids (scale bar = 100 µm); Video S1: Confocal imaging of 3D structures of a single trophoblastic organoid stained against KRT18; Video S2: Time-lapse imaging (6 days) of a single endometrial organoid showing its rotatory movement around its axis.

## Abbreviations

The following abbreviations are frequently used in this manuscript:

TOs	Trophoblast organoids
ICSI	Intracytoplasmic sperm injection
EOs	Endometrial organoids
3D	Three-dimensional
PBS	Phosphate-buffered saline
TLCs	Trophoblastic lacunoids/cystoids

## References

[1] Burton G.J., Jauniaux E. (2015). What is the placenta?. Am. J. Obs. Gynecol..

[2] Turco M.Y., Gardner L., Kay R.G., Hamilton R.S., Prater M., Hollinshead M.S., McWhinnie A., Esposito L., Fernando R., Skelton H. (2018). Trophoblast organoids as a model for maternal–fetal interactions during human placentation. Nature.

[3] Saadeldin I.M., Ehab S., Noreldin A.E., Swelum A.A.-A., Bang S., Kim H., Yoon K.Y., Lee S., Cho J. (2024). Current strategies using 3D organoids to establish in vitro maternal-embryonic interaction. J. Vet. Sci..

[4] Haider S., Meinhardt G., Saleh L., Kunihs V., Gamperl M., Kaindl U., Ellinger A., Burkard T.R., Fiala C., Pollheimer J. (2018). Self-renewing trophoblast organoids recapitulate the developmental program of the early human placenta. Stem Cell Rep..

[5] Pascual F. (2022). Trophoblast Organoids: A New Tool for Studying Placental Development. Environ. Health Perspect..

[6] Vento-Tormo R., Efremova M., Botting R.A., Turco M.Y., Vento-Tormo M., Meyer K.B., Park J.-E., Stephenson E., Polański K., Goncalves A. (2018). Single-cell reconstruction of the early maternal–fetal interface in humans. Nature.

[7] Arutyunyan A., Roberts K., Troule K., Wong F.C.K., Sheridan M.A., Kats I., Garcia-Alonso L., Velten B., Hoo R., Ruiz-Morales E.R. (2023). Spatial multiomics map of trophoblast development in early pregnancy. Nature.

[8] Sheridan M.A., Fernando R.C., Gardner L., Hollinshead M.S., Burton G.J., Moffett A., Turco M.Y. (2020). Establishment and differentiation of long-term trophoblast organoid cultures from the human placenta. Nat. Protoc..

[9] Yang L., Liang P., Yang H., Coyne C.B. (2024). Trophoblast organoids with physiological polarity model placental structure and function. J. Cell Sci..

[10] Wu H., Huang X.Y., Sun M.X., Wang Y., Zhou H.Y., Tian Y., He B., Li K., Li D.Y., Wu A.P. (2023). Zika virus targets human trophoblast stem cells and prevents syncytialization in placental trophoblast organoids. Nat. Commun..

[11] Li X., Li Z.H., Wang Y.X., Liu T.H. (2023). A comprehensive review of human trophoblast fusion models: Recent developments and challenges. Cell Death Discov..

[12] Shibata S., Kobayashi E.H., Kobayashi N., Oike A., Okae H., Arima T. (2020). Unique features and emerging in vitro models of human placental development. Reprod. Med. Biol..

[13] Horii M., Touma O., Bui T., Parast M.M. (2020). Modeling human trophoblast, the placental epithelium at the maternal fetal interface. Reproduction.

[14] Saadeldin I.M., Han A., Bang S., Kang H., Kim H., Abady M.M., Jeong J.-S., Kwon H.-J., Lee S., Cho J. (2023). Generation of porcine endometrial organoids and their use as a model for enhancing embryonic attachment and elongation. Reproduction.

[15] Moffett A. (2023). Organoid cultures as model systems to study disorders of the human endometrium and placenta. Obstet. Gynaecol. Reprod. Med..

[16] Wen L., Tang F. (2022). Organoid research on human early development and beyond. Med. Rev..

[17] Enders A.C. (1995). Transition from Lacunar to Villous Stage of Implantation in the Macaque, Including Establishment of the Trophoblastic Shell. Acta Anat..

[18] Charles A.K., Faye-Petersen O.M. (2014). Human Placental Development from Conception to Term. Pathobiology of Human Disease.

[19] Leiser R., Beier H.M. (1988). Morphological Studies of Lacunar Formation in the Early Rabbit Placenta. Placental Vascularization and Blood Flow.

[20] Saadeldin I.M., Swelum A.A.-A., Elsafadi M., Mahmood A., Yaqoob S.H., Alfayez M., Alowaimer A.N. (2019). Effects of *all-trans* retinoic acid on the in vitro maturation of camel (*Camelus dromedarius*) cumulus-oocyte complexes. J. Reprod. Dev..

[21] Ebrahimi M., Mara L., Chessa B., Chessa F., Parham A., Dattena M. (2021). Optimizing injection time of GFP plasmid into sheep zygote. Reprod. Domest. Anim..

[22] Swelum A.A., Saadeldin I.M., Bahadi M., Afifi M., Al-Mutary M., Alowaimer A.N. (2018). The effect of heterologous seminal plasma from ram, buck or camel on the freezability of ram semen. Vet. Med..

[23] Ressaissi Y., Anzalone D.A., Palazzese L., Czernik M., Loi P. (2021). The impaired development of sheep ICSI derived embryos is not related to centriole dysfunction. Theriogenology.

[24] Saadeldin I.M., Kim B., Lee B., Jang G. (2011). Effect of different culture media on the temporal gene expression in the bovine developing embryos. Theriogenology.

[25] Saadeldin I.M., Kim S.J., Lee B.C. (2015). Blastomeres aggregation as an efficient alternative for trophoblast culture from porcine parthenogenetic embryos. Dev. Growth Differ..

[26] Saadeldin I.M., Swelum A.A.-A., Elsafadi M., Moumen A.F., Alzahrani F.A., Mahmood A., Alfayez M., Alowaimer A.N. (2017). Isolation and characterization of the trophectoderm from the Arabian camel (*Camelus dromedarius*). Placenta.

[27] Saadeldin I.M., Kim S.J., Choi Y.B., Lee B.C. (2014). Post-maturation zona perforation improves porcine parthenogenetic trophoblast culture. Placenta.

[28] Ralston A., Cox B.J., Nishioka N., Sasaki H., Chea E., Rugg-Gunn P., Guo G., Robson P., Draper J.S., Rossant J. (2010). Gata3 regulates trophoblast development downstream of Tead4 and in parallel to Cdx2. Development.

[29] Home P., Ray S., Dutta D., Bronshteyn I., Larson M., Paul S. (2009). GATA3 Is Selectively Expressed in the Trophectoderm of Peri-implantation Embryo and Directly Regulates Cdx2 Gene Expression. J. Biol. Chem..

[30] Pötgens A.J.G., Drewlo S., Kokozidou M., Kaufmann P. (2004). Syncytin: The major regulator of trophoblast fusion? Recent developments and hypotheses on its action. Hum. Reprod. Update.

[31] Liang X., Qiu X., Ma Y., Xu W., Chen S., Zhang P., Liu M., Lin X. (2023). KRT18 regulates trophoblast cell migration and invasion which are essential for embryo implantation. Reprod. Biol. Endocrinol..

[32] Siriwardena D., Munger C., Penfold C., Kohler T.N., Weberling A., Linneberg-Agerholm M., Slatery E., Ellermann A.L., Bergmann S., Clark S.J. (2024). Marmoset and human trophoblast stem cells differ in signaling requirements and recapitulate divergent modes of trophoblast invasion. Cell Stem Cell.

[33] Han Q., Zheng L., Liu Z., Luo J., Chen R., Yan J. (2018). Expression of β-catenin in human trophoblast and its role in placenta accreta and placenta previa. J. Int. Med. Res..

[34] Pera M., Keramari M., Razavi J., Ingman K.A., Patsch C., Edenhofer F., Ward C.M., Kimber S.J. (2010). Sox2 Is Essential for Formation of Trophectoderm in the Preimplantation Embryo. PLoS ONE.

[35] Niakan K.K., Eggan K. (2013). Analysis of human embryos from zygote to blastocyst reveals distinct gene expression patterns relative to the mouse. Dev. Biol..

[36] Mathew D.J., Peterson K.D., Senn L.K., Oliver M.A., Ealy A.D. (2022). Ruminant conceptus-maternal interactions: Interferon-tau and beyond. J. Anim. Sci..

[37] Bott R.C., Ashley R.L., Henkes L.E., Antoniazzi A.Q., Bruemmer J.E., Niswender G.D., Bazer F.W., Spencer T.E., Smirnova N.P., Anthony R.V. (2010). Uterine Vein Infusion of Interferon Tau (IFNT) Extends Luteal Life Span in Ewes1. Biol. Reprod..

[38] Bazer F.W., Ott T.L., Spencer T.E. (1994). Pregnancy recognition in ruminants, pigs and horses: Signals from the trophoblast. Theriogenology.

[39] Farin C.E., Imakawa K., Hansen T.R., McDonnell J.J., Murphy C.N., Farin P.W., Roberts R.M. (1990). Expression of Trophoblastic Interferon Genes in Sheep and Cattle1. Biol. Reprod..

[40] Molbay M., Kipmen-Korgun D., Korkmaz G., Ozekinci M., Turkay Korgun E. (2018). Human Trophoblast Progenitor Cells Express and Release Angiogenic Factors. Int. J. Mol. Cell. Med..

[41] Chung I.B., Yelian F.D., Zaher F.M., Gonik B., Evans M.I., Diamond M.P., Svinarich D.M. (2000). Expression and regulation of vascular endothelial growth factor in a first trimester trophoblast cell line. Placenta.

[42] Enders A.C., King B.F. (1991). Early stages of trophoblastic invasion of the maternal vascular system during implantation in the macaque and baboon. Am. J. Anat..

[43] Enders A.C., Lantz K.C., Peterson P.E., Hendrickx A.G. (1997). From blastocyst to placenta: The morphology of implantation in the baboon. Hum. Reprod. Update.

[44] Bills V.L., Hamdollah-Zadeh M., Soothill P.W., Harper S.J., Bates D.O. (2014). The role of VEGF-A165b in trophoblast survival. BMC Pregnancy Childbirth.

[45] Fan X., Muruganandan S., Shallie P.D., Dhal S., Petitt M., Nayak N.R. (2021). VEGF Maintains Maternal Vascular Space Homeostasis in the Mouse Placenta through Modulation of Trophoblast Giant Cell Functions. Biomolecules.

[46] Ghosh D., Sharkey A.M., Charnock-Jones D.S., Dhawan L., Dhara S., Smith S.K., Sengupta J. (2000). Expression of vascular endothelial growth factor (VEGF) and placental growth factor (PlGF) in conceptus and endometrium during implantation in the rhesus monkey. Mol. Hum. Reprod..

[47] Zhang Z., Wang X., Zhang L., Shi Y., Wang J., Yan H. (2017). Wnt/β-catenin signaling pathway in trophoblasts and abnormal activation in preeclampsia. Mol. Med. Rep..

[48] Kiewisz J., Wasniewski T., Kmiec Z. (2015). Participation of WNT andβ-Catenin in Physiological and Pathological Endometrial Changes: Association with Angiogenesis. BioMed Res. Int..

[49] Yu N., Wu J.-L., Xiao J., Fan L., Chen S.-H., Li W. (2019). HIF-1α regulates angiogenesis via Notch1/STAT3/ETBR pathway in trophoblastic cells. Cell Cycle.

[50] Taghizadeh E., Tazik K., Taheri F., Shayankia G., Gheibihayat S.M., Saberi A. (2022). Abnormal angiogenesis associated with HIF-1α/VEGF signaling pathway in recurrent miscarriage along with therapeutic goals. Gene Rep..

[51] Suwińska A., Czołowska R., Ożdżeński W., Tarkowski A.K. (2008). Blastomeres of the mouse embryo lose totipotency after the fifth cleavage division: Expression of Cdx2 and Oct4 and developmental potential of inner and outer blastomeres of 16- and 32-cell embryos. Dev. Biol..

[52] Saadeldin I.M., Koo O.J., Kang J.T., Kwon D.K., Park S.J., Kim S.J., Moon J.H., Oh H.J., Jang G., Lee B.C. (2012). Paradoxical effects of kisspeptin: It enhances oocyte in vitro maturation but has an adverse impact on hatched blastocysts during in vitro culture. Reprod. Fertil. Dev..

[53] Cecchele A., Cermisoni G.C., Giacomini E., Pinna M., Vigano P. (2022). Cellular and Molecular Nature of Fragmentation of Human Embryos. Int. J. Mol. Sci..

[54] Lee Y.-J., Lin Y.-P., Cheng E.-H., Chen C.-H., Huang C.-C., Lin P.-Y., Lee T.-H., Lee M.-S. (2023). The presence of vacuoles in blastocysts is negatively associated with euploidy and live birth rates. Fertil. Steril..

[55] Saraste A. (1999). Morphologic criteria and detection of apoptosis. Herz.

[56] Otun H.A., Lash G.E., Innes B.A., Bulmer J.N., Naruse K., Hannon T., Searle R.F., Robson S.C. (2011). Effect of tumour necrosis factor-α in combination with interferon-γ on first trimester extravillous trophoblast invasion. J. Reprod. Immunol..

[57] Moindjie H., Santos E.D., Gouesse R.-J., Swierkowski-Blanchard N., Serazin V., Barnea E.R., Vialard F., Dieudonné M.-N. (2016). Preimplantation factor is an anti-apoptotic effector in human trophoblasts involving p53 signaling pathway. Cell Death Dis..

[58] Xing F., Kong L., Chen S. (1999). Apoptosis of cultured human trophoblasts induced by tumor necrosis factor-alpha and interferon-gamma. Zhonghua Fu Chan Ke Za Zhi.

[59] Yamauchi H., Katayama K.-I., Ueno M., He X.J., Mikami T., Uetsuka K., Doi K., Nakayama H. (2007). Essential role of p53 in trophoblastic apoptosis induced in the developing rodent placenta by treatment with a DNA-damaging agent. Apoptosis.

[60] Oldak B., Wildschutz E., Bondarenko V., Comar M.-Y., Zhao C., Aguilera-Castrejon A., Tarazi S., Viukov S., Pham T.X.A., Ashouokhi S. (2023). Complete human day 14 post-implantation embryo models from naive ES cells. Nature.

[61] Shahbazi M.N., Jedrusik A., Vuoristo S., Recher G., Hupalowska A., Bolton V., Fogarty N.M.E., Campbell A., Devito L.G., Ilic D. (2016). Self-organization of the human embryo in the absence of maternal tissues. Nat. Cell Biol..

[62] Alexandrova M., Manchorova D., You Y., Mor G., Dimitrova V., Dimova T. (2022). Functional HLA-C expressing trophoblast spheroids as a model to study placental–maternal immune interactions during human implantation. Sci. Rep..

[63] Tachi S., Tachi C., Lindner H.R. (1970). Ultrastructural Features of Blastocyst Attachment and Trophoblastic Invasion in the Rat. Reproduction.

[64] Potts M., Psychoyos A. (1967). Evolution of the ultrastructure of the ovoendometrial connections under the influence of estrogen in the rat during experimental retardation of nidation. C R Acad. Hebd. Seances Acad. Sci. D.

[65] Potts D.M. (1968). The ultrastructure of implantation in the mouse. J. Anat..

[66] Zhai J., Xiao Z., Wang Y., Wang H. (2022). Human embryonic development: From peri-implantation to gastrulation. Trends Cell Biol..

[67] Enders A.C. (2007). Implantation in the Macaque: Expansion of the Implantation Site During the First Week of Implantation. Placenta.

[68] Enders A.C., Lopata A. (1999). Implantation in the marmoset monkey: Expansion of the early implantation site. Anat. Rec..

[69] Seo H., Bazer F.W., Burghardt R.C., Johnson G.A. (2019). Immunohistochemical Examination of Trophoblast Syncytialization during Early Placentation in Sheep. Int. J. Mol. Sci..

