# Supplementary material for: Modeling Early Stages of Trophectoderm–Endometrium Interactions Using Trophoblastic and Endometrial Organoids and the Generation of Lacunoids/Cystoids
Source: Cells. 2025 Jul 9;14(14):1051. doi: 10.3390/cells14141051 (PMC12293716; doi:10.3390/cells14141051)
Supplement: Supplementary file 1 [file cells-14-01051-s001.zip › Supplementary Table S1.pdf]

Table.

Gene name	Accession #	Forward (5' → 3')	Reverse (5' → 3')	Size (bp)
IFNT	XM_027963945.1	CCCCATTGTGACCGTGAAGA	CTCCACTCTGACGGTTTCCC	97
CDX2	XM_027973894.2	GGCAGCCAAGTGAAAACCAG	TGAAACCAAATTTTAACCTGCCTCT	173
POU5F1 (OCT4)	FJ970649.1	GGTGGAGGAAGCTGACAACA	TGCCTCTCACCTTGTTCTCG	110
SOX17	XM_027972959.3	AGCAAGATGCTGGGCAAGT	TTGGGATGGTCCTGCATGTG	107
SOX2	NM_001318074.1	GCAGACCTACATGAACGGCT	CGGGGAGGTACATGCTGATC	200
VIM	JX534524.1	CAGGCCCAGATTCAAGAGCA	ACGCTCTCATACTGCTGACG	101
VEGFA	NM_001025110.1	GCTCTCTTGGGTGCATTGGA	GCCTGGGACCACTTGGC	66
VEGFR1	AF513910.1	CTTTCAGGCTCGGAGGAGAT	GGCATCACGGAAGTGTATCCA	75
VEGFR2 (KDR)	NM_001278565.2	GGCATCACGGAAGTGTATCCA	CGTTCTTTTCCGACAAGAGGA	65
TP53	NM_001009403.1	TTCTTCGTCTGCTCAGCCTG	GCTTCTGACCTCTCCCGAAC	140
TNFAIP6	XM_004004704.4	TATGGGAAGAGGCTCACGGA	TTCTGGCTGCCTCTAACTGC	195
ACTB	NM_001009784.3	AAGTACCCCATTGAGCACGG	CATCTTCTCACGGTTGGCCT	156
GAPDH	NM_001190390.1	ACAGTCAAGGCAGAGAACGG	GACTCCACCACGTACTCAGC	119

TNFAIP6 is an inflammation-associated protein that plays an important role in extracellular matrix formation and cell migration
