# Supplementary figures and images for: Modeling Early Stages of Trophectoderm–Endometrium Interactions Using Trophoblastic and Endometrial Organoids and the Generation of Lacunoids/Cystoids
Source: Cells. 2025 Jul 9;14(14):1051. doi: 10.3390/cells14141051 (PMC12293716; doi:10.3390/cells14141051)

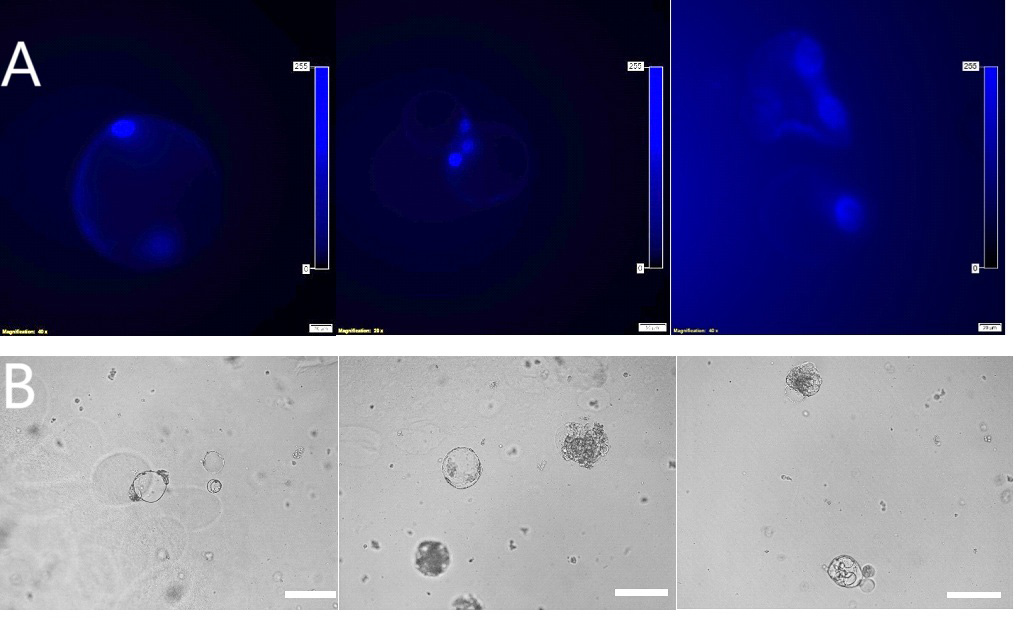

Supplement: Supplementary file 1 [file cells-14-01051-s001.zip › Supplementary Figure S1.jpg]

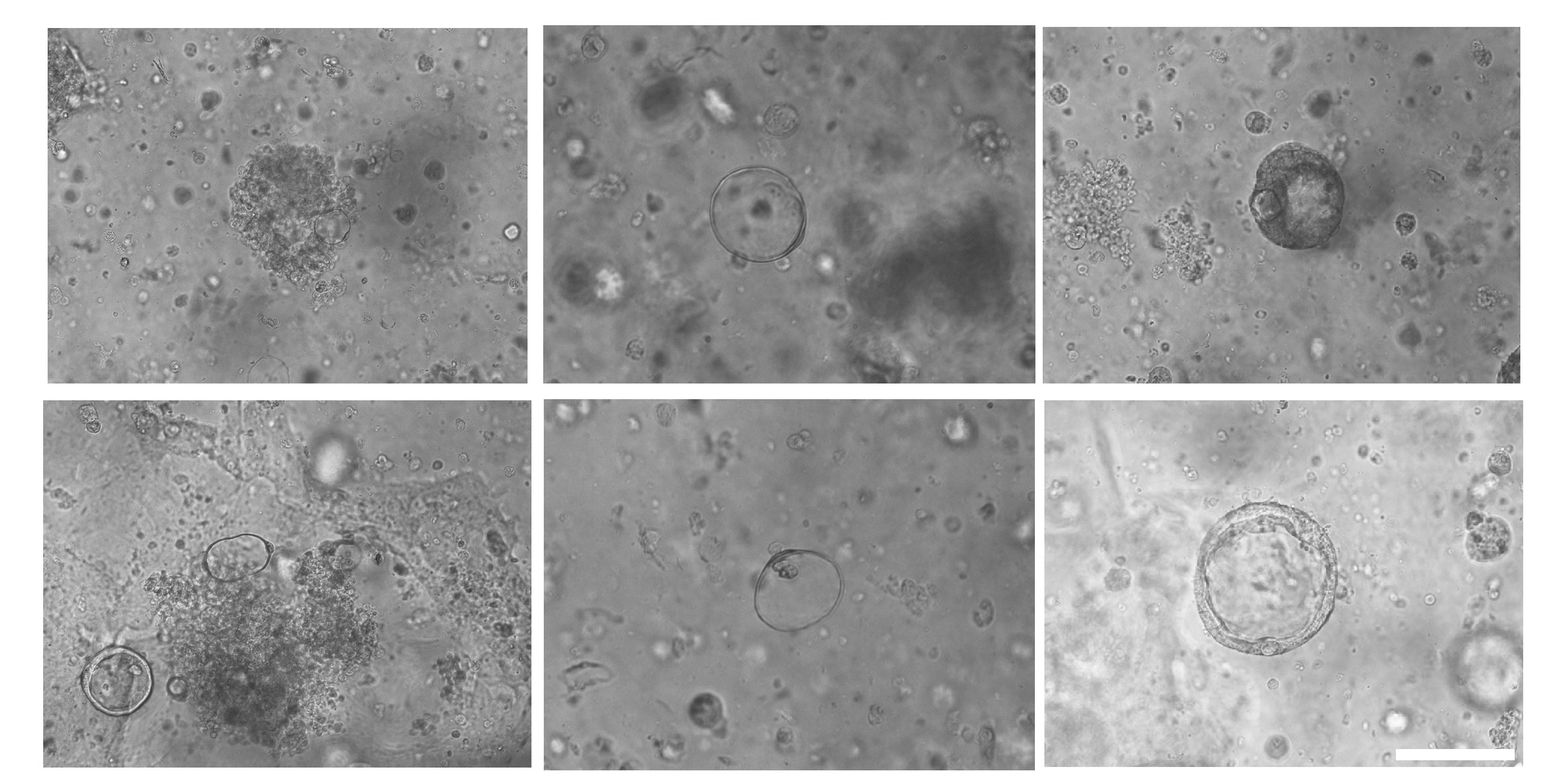

Supplement: Supplementary file 1 [file cells-14-01051-s001.zip › Supplementary Figure S2.jpg]
